# Proximity and Same Case Marking Do Not Increase Attraction Effect in Comprehension: Evidence From Eye-Tracking Experiments in Korean

**DOI:** 10.3389/fpsyg.2019.01320

**Published:** 2019-06-06

**Authors:** Nayoung Kwon, Patrick Sturt

**Affiliations:** ^1^Department of English, Konkuk University, Seoul, South Korea; ^2^Department of Psychology, University of Edinburgh, Edinburgh, United Kingdom

**Keywords:** Korean, attraction, honorific agreement, subject-verb agreement, eye tracking, proximity, case marking, memory representation

## Abstract

Previous studies have suggested that during the on-line sentence processing, relevant memory representations are directly accessed based on cues at retrieval (McElree et al., 2003). Under this hypothesis, retrieval cues activate any memory representation with matching features, leading to the so-called attraction effect. This predicts that attraction effects would be modulated by memory representation of a distractor. Here, we investigated this possibility, focusing on two factors (i.e., proximity to the retrieval point and the number of matching features) that would affect representation of a distractor in three Korean eye-tracking experiments. We predicted that if memory representation of a distractor decays over time, a distractor close to a retrieval point would lead to stronger attraction effects. We also predicted that a distractor would be more likely to lead to interference when it shares a higher number of matching features with the retrieval cues of a dependency, relative to the target of the dependency, due to multiple direct accesses based on multiple matching cues. However, the results did not show evidence that proximity of a distractor to the retrieval point enhanced attraction effects. Likewise, there was no evidence that a greater number of matching cues of a distractor alone would trigger more mis-retrieval, in contrast to a previous finding that a greater number of mismatching cues of a licit antecedent in addition to a greater number of matching cues of a distractor did so (Parker and Phillips, 2017). On the other hand, the results suggested that a distractor marked with nominative case was more likely to be mis-retrieved as the subject of a verb, compared to a distractor marked with a dative case, suggesting that the subject grammatical role is a critical cue for a subject-verb agreement. These results are best compatible with the hypothesis that retrieval cues are weighted, possibly depending on the nature of the dependency that is currently processed.

## Introduction

Successful processing of a long-distance dependency requires retrieval of linguistic items in working memory. For example, in (1) the head NP (i.e., *the book*) of a relative clause should be retrieved at the embedded verb (i.e., *admired*), where it can be associated with a thematic role within the relative clause, and in (2) the head NP (i.e., *the key*) of a complex NP should be retrieved at the main verb (i.e., *was*) to form a subject-verb agreement dependency.

(1) This was the book that the editor admired.
(2) The key to the cabinet was rusty from many years of disuse.

Results of previous studies have been argued to support a content-addressable direct access model of retrieval (McElree, 2000; McElree et al., 2003; c.f., Kintsch, 1970), according to which items stored in working memory are activated in parallel, based on the matching of retrieval cues (see Lewis and Vasishth, 2005) for an implementation of such a content-addressable cue-based retrieval model of sentence processing).

Importantly to the goal of the study, this cue-based parallel retrieval mechanism predicts that the processing of a linguistic dependency can be affected by elements in memory that are not licit parts of that dependency. This arises because the model predicts that any item in memory that matches in features with a retrieval cue will be activated to a certain extent, even when the feature match is only partial. Such effects have been discussed in terms of both inhibitory and facilitatory mechanisms. For inhibitory effects, it has been shown that when NPs (noun phrases) in memory are of the same type, this can result in increased processing costs, and this effect has been argued to be due to partial activation of the illicit NP (similarity-based interference: Gordon et al., 2001). Thus, as Lee et al. (2006) discussed, rereading times of NP1 and NP2 in sentences of the form of (3) were longer after reading the verb region (i.e., retrieval point) when they were both descriptive nouns than when they were of different types (e.g., descriptive NP1 and a pronominal NP2), probably due to enhanced difficulty in establishing legitimate syntactic or semantic relations between NPs and the verbs. These results suggest that retrieval cues activated all memory representations of linguistic items with matching features in parallel and that the mis-retrieved NP interfered with the processing of a subject-verb dependency.

(3) Two NPs with the nominative caser marker
[_MAINCLAUSE_ subject NP_1_ [_EMBEDDEDCLAUSE_ subject NP_2_ _EMB_verb] _MAIN_verb].

Turning to facilitatory effects, the so-called *attraction* effect is a case where the activation of an illicit element has been argued to lead to mis-retrieval of that element instead of the target on a proportion of trials, leading to an overall facilitation of processing (Lewis and Vasishth, 2005; Wagers et al., 2009). The attraction effect is a “grammatical illusion” where processing difficulty due to ungrammaticality is reduced when a feature-matching distractor is mis-retrieved. For example, sentences in (c) and (d) in (4) are both ungrammatical as the licit subject (*the key*) mismatches the verb (*were*) in its number feature, but they differ from each other in that sentence (d) has a distractor NP with a plural number feature (e.g., *the cabinets…were*) while sentence (c) does not (e.g., *the cabinet…were*).

(4) Experimental sentences in Pearlmutter et al. (1999)
  - The key to the cabinet was rusty from many years of disuse.
  - The key to the cabinets was rusty from many years of disuse.
  ^*^The key to the cabinet were rusty from many years of disuse.
  ^*^The key to the cabinets were rusty from many years of disuse.

If processing difficulty of the subject-verb agreement in sentences like (4) is only affected by the licit subject (i.e., *the key*), then an equal level of processing difficulty is predicted for sentences (c) and (d) in comparison to their grammatical counterpart sentences (a) and (b), regardless of different number features of a distractor in (c) and (d). However, experimental results have shown reduced processing difficulty sentences like (d) in comparison to sentences like (c), suggesting that the processing difficulty of the subject-verb agreement is also affected by a distractor item which does not participate in the dependency (see also Nicol et al., 1997; Pearlmutter et al., 1999; Thornton and MacDonald, 2003; Drenhaus et al., 2005; Vasishth et al., 2008; Xiang et al., 2009; Dillon et al., 2013; Tanner et al., 2014; Lago et al., 2015; Parker and Phillips, 2017; for related effects in production, see Bock and Miller, 1991; Bock and Cutting, 1992; Bock and Eberhard, 1993; Vigliocco and Nicol, 1998; Hartsuiker et al., 2001; Haskell and MacDonald, 2003; Thornton and MacDonald, 2003; for related inhibitory effects, see Lewis, 1996; Gordon et al., 2001, 2004, 2006).

Similar effects have been found in Korean as well. Although Korean does not have rich verbal agreement, and so verbs in Korean do not agree with their subjects in person or number in most cases (Sohn, 1999), the subject honorific marker –*si* is an exception to this. –*Si* attaches to the stem of a verb, marking the speaker's respect for its agreeing local subject as in (5). Accordingly, –*si* can only occur with a honorifiable NP such as *grandpa, uncle* or *teacher* but not with nouns such as *kid* or *burglar*, which would be regarded as denoting people who are low in their perceived social status as shown in (6). In certain aspects, the –*si* subject-verb agreement in Korean is different from the number or person subject-verb agreement in English, given that –*si* agreement is pragmatically motivated, and given that the use of –*si* is optional such that its omission does not render the sentence unacceptable as shown in (5). Yet, in a previous study, Kwon and Sturt (2016b) showed that retrieval processes underlying the processing of –*si* agreement in Korean are similar to those of the number or person subject-verb agreement in English. Thus, in a sentence where a second NP forms a dependency with an embedded verb as in (7), a distractor with a matching honorific feature (e.g., chair) has been shown to reduce the processing difficulty due to honorific feature mismatches between a verb and its licit subject (e.g., embedded verb with –*si* and *Inho*, personal names in Korean, without honorific feature; Kwon and Sturt, 2016b).


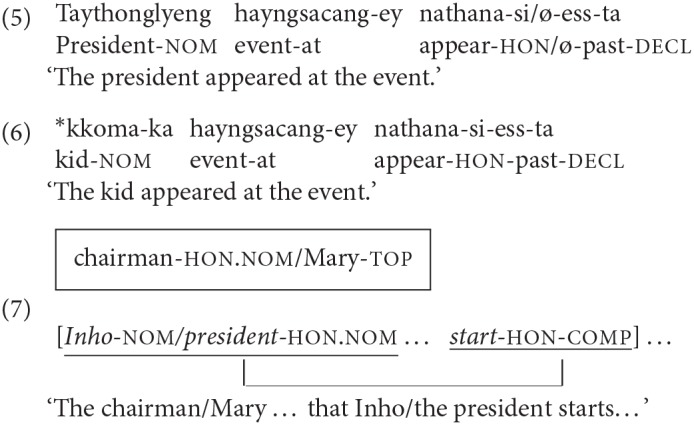


In many cases, attraction effects have been found in ungrammatical sentences at a relatively late processing stage after the ungrammaticality of sentences is detected (Pearlmutter et al., 1999; Wagers et al., 2009; Dillon et al., 2013; Lago et al., 2015; cf. Van Dyke, 2007). Given this, and given the fact that these studies did not find reliable evidence of attraction for grammatical sentences, it has been proposed that the attraction effects arise as an error-driven process, whereby distractors are retrieved as a repair strategy (cf. Wagers et al., 2009). However, the effect of a distractor found in Kwon and Sturt (2016b) is only partially compatible with this proposal. While the grammaticality effect (i.e., cost of ungrammatical relative to grammatical conditions) indeed preceded the effect of a distractor in the eye-movement record, the effect of the distractor was not limited to ungrammatical sentences. Instead, the processing of grammatical sentences was also affected by a distractor, leading to longer reading times when the distractor (e.g., Mary without honorific feature) mismatched the embedded verb in honorific features. These results were taken to suggest that the attraction effect is not an error-driven mechanism. Instead, Kwon and Sturt proposed that the effect of a distractor is likely to result from general working memory principles of activating of potential items in memory (see also Vasishth et al., 2008). Note, however, that the effect observed by Kwon and Sturt showed a facilitation for grammatical sentences where the distractor matched the honorific features of the verb, while the model proposed by Lewis and Vasishth (2005) would have predicted inhibition in this condition due to similarity-based interference. We return to this issue in the discussion.

Despite some differences in the interpretation, these results suggest the possibility that the processing of the retrieval process is modulated by memory representations of a distractor as well as that of a licit target item. For example, while a larger pool of items in memory does not affect the retrieval speed of a target item (McElree, 2000), assuming that retrieval of a linguistic item in memory is preceded by the reactivation of its memory representation which decays over time, it is possible that a distractor is more easily activated and thus interferes more with the processing of a dependency when it is closer to a retrieval point than when it is further away (Lewis and Vasishth, 2005)^1^. In fact, manipulating the linear distance between the subject and the verb as well as the number feature of the subject and its intervening object, Kaan (2002) showed that participants better remembered the number features of the licit subject when the linear distance of the subject and the verb was shorter, suggesting the relevance of linear distance in the context of retrieval processes. Likewise, if retrieval is based on a parallel feature matching process, it is also possible that a distractor is more prone to affect retrieval of a target item when it has a larger number of matching features than when it has fewer. Supporting evidence for this latter observation comes from a study on the processing of reflexives, reported by Parker and Phillips (2017). Previous studies had shown that unlike number/person subject-verb agreement or negative polarity items, the processing of reflexives is not easily affected by a distractor (Sturt, 2003; Xiang et al., 2009; Dillon et al., 2013). For example, in Sturt's (2003) study of the processing of reflexives in sentences like (8), readers slowed down in the early parsing stages only when the licit subject (*He/She*) mismatched the reflexive in gender features. On the other hand, the gender feature of the intervening distractor (*the surgeon*) did not affect the processing until later processing stages, during the final interpretation (Sturt, 2003). However, Parker and Phillips (2017) showed that, when a distractor is associated with a larger number of matching (and probably semantically more salient) features [i.e., animacy as well as gender features in (9)], the processing of an emphatic reflexive is affected by a distractor in a similar manner to that of number/person agreement. Thus, the processing difficulty due to animacy/gender mismatches in a dependency involving a reflexive (e.g., the discovery-himself) was significantly reduced when there was a feature matching distractor (e.g., the researcher). These results suggest a possibility that retrieval is sensitive to memory representations of a distractor.

(8) He/She remembered that the surgeon had pricked himself/herself with a used syringe.
(9) The doctor/discovery that the researcher/report described meticulously was certified after debunking the urban myth himself in the new scientific journal.

Thus, in the experiments described below, we aimed to further investigate the effect of memory representations of a distractor on retrieval. We examined the effect of proximity by manipulating the linear distance of a distractor to a retrieval point, and the effect of the degree of similarity by manipulating the number of matching features between retrieval cues and a distractor, across the three experiments. To this aim, we ran eye-tracking studies of the processing of a subject-verb –*si* agreement using subject control, object control, and center-embedded clause constructions in Korean. The target stimuli of Experiments 1 through 3 are schematically presented in sentences (10), (11), and (12) respectively. As illustrated in these sentences, the structurally legitimate subject NP for the embedded verb differs depending on the sentence type. For example, in the subject control construction in Experiment 1, NP1 is the licit subject for the embedded verb, while NP2 is the licit subject in the object control construction in Experiment 2 and in the center-embedded construction in Experiment 3. Thus, in Experiment 1, NP2 is a distractor (i.e., NP in the dotted square), while in Experiments 2 and 3, NP1 is. Accordingly, the distractor linearly intervenes in the subject-verb dependency in Experiment 1 but it does not in Experiment 2 or Experiment 3. Thus, the comparison of the results of Experiment 1 and 2 could reveal the effect of proximity of a distractor to its retrieval point. If a distractor whose memory representations are more highly activated is more likely to lead to interference or attraction, then it can be predicted that an effect of a distractor will be stronger in Experiment 1 than in Experiment 2.


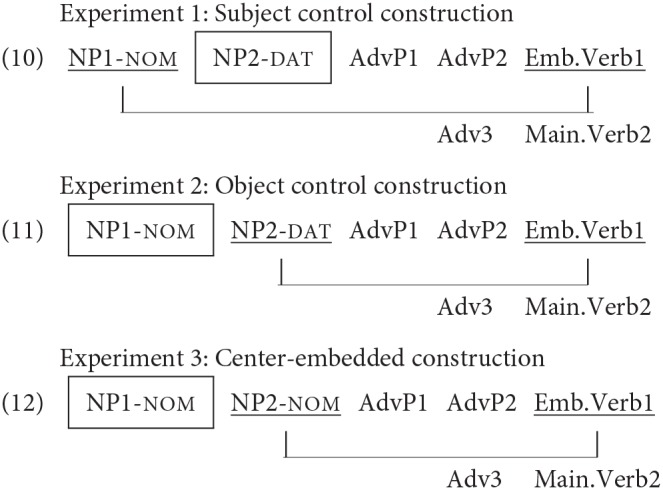


On the other hand, the comparison of Experiments 2 and 3 could reveal the effect of the number of matching features of a distractor. Previous production studies have shown that similarity in case marking of the licit subject and a distractor led to increased attraction effects (Hartsuiker et al., 2001). Likewise, assuming that case information is a retrieval cue in comprehension, the morphology on the verb in Experiment 2 encodes a retrieval cue of dative case for its (overt) subject argument, while in Experiment 3, the retrieval cue is for nominative case. Thus, the distractor in Experiment 3 has more retrieval cue features than the distractor in Experiments 1 or 2. If a greater degree of feature overlap between distractor and retrieval cues leads to greater interference or attraction, then it can be predicted that the effect of the distractor will be stronger in Experiment 3, where the distractor matches the case retrieval cue, than in Experiment 2, where it does not. Thus, comparison of the general patterns of the results of these experiments would further our understanding of how memory representations of a distractor affect the retrieval processes, leading to a fuller understanding of the mechanisms underlying the retrieval processes in general. Below, we first report these three eye-tracking experiments, and then go on to present cross-experiment comparisons to address these questions.

## Experiment 1

Experiment 1 investigated the processing of subject-verb honorific agreement in the subject control construction with the suffix –*keyss* “will” or “plan to” in Korean. In this particular construction, as used in Experiment 1, the embedded verb, marked with –*keyss*, indicates that the matrix subject (NP1) is the controller for PRO, as shown in (13). The construction is roughly translated as the subject “planning to” execute the action predicated in the embedded verb. Thus, the dependency is formed between NP1 and the embedded verb (via PRO), as illustrated in (10) above. On the other hand, the dative marked indirect object cannot serve as a controller although it is linearly closer to the embedded verb, intervening with the subject-verb honorific agreement.

(13) Subject control construction used in Experiment 1
[NP1*_*i*_*-nom NP2_*k*_-dat
[PRO*_*i*_* leave-hon-subject.ctrl-decl-comp] said]
‘NP_*i*_ told NP2_*k*_ that PRO${}_{i{/}^{*}k}$ will leave.'

Personal names in Korean do not have honorific features. Thus, following Kwon and Sturt (2016b), to investigate the memory retrieval processes underlying the processing of a dependency, we manipulated the honorific features of NP1 and NP2 of the experimental sentences, by using either personal names (i.e., NH: non-honorifiable) or descriptive NPs (i.e., H: honorifiable)^2^. On the other hand, the embedded verb is always marked with an honorific marker –*si* (see R5 in Table 1 below). Accordingly, there are two congruous conditions (i.e., H_NP1_-NH_NP2_ and H_NP1_-H_NP2_) and two incongruous conditions (i.e., NH_NP1_-H_NP2_ and NH_NP1_-NH_NP2_), as shown in Table 1.

**Table 1:**
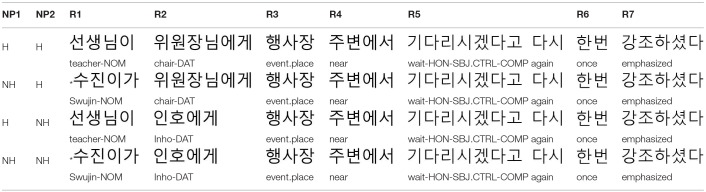
Example experiment item in Experiment 1: Subject control construction.

*The teacher_i_ /Swujin_i_ emphasized to the chair/Indo that ___i_ would wait near the event place*.

Since the honorific suffix –*si* should agree with the verb's subject in Korean, we predict that mismatched honorific features of the incongruous conditions with the NH subject (NH-H and NH-NH conditions) will incur processing difficulty (Kwon and Sturt, 2016b). Thus, the incongruous conditions will show longer reading times at the embedded verb marked with –*si*– when compared with their congruous counterpart sentences with the H subject (H-H and H-NH conditions). Crucially, although control information is accessed early during on-line sentence processing (Kwon and Sturt, 2014, 2016a), it has been also shown that the processing of subject-verb honorific agreement can be affected by a feature-matching yet structurally illicit distractor in a control construction (Sturt and Kwon, 2015). If so, we predict that the reading time penalty for the incongruous condition will be reduced in the NH-H condition, where the distractor matches the honorific features of the verb, compared to the NH-NH condition, where it does not, resulting in an interaction between the honorific features of the subject and the dative object at the embedded verb position. This interactive pattern would be consistent with previous studies investigating subject-verb number agreement in English (e.g., Wagers et al., 2009; Dillon et al., 2013).

### Participants

Twenty eight native speakers of Korean (mean age: 23.46; range 19–27) participated in the study. At the time of the experiment, they were all either undergraduate or graduate students at Konkuk University, Korea and received KRW 10,000 per hour for their participation. They all had normal or corrected-to-normal vision. Written informed consent was obtained from all the participants.

### Materials

Forty sets of experimental sentences like those in Table 1 were created. All the experimental sentences contained two NPs with a + or – value for the honorific feature. On the other hand, the embedded verb was always marked with –*si*, and this verb formed a dependency with one of these NPs.

Before the main experiment, we first conducted a norming study to control for the plausibility of the event described with an embedded verb with NP1 or NP2 as a potential subject. For example, for the sentences in Table 1 four norming sentences were created using NP1 or NP2 (H_NP1_, NH_NP1_, H_NP2_, NH_NP2_) and the embedded verb, where the verbs of the norming sentences were matched with these NPs in their honorific features, as shown in (14) and (15). Thirty-two native Korean speakers participated in the norming study, each receiving KRW 3,000. At the time of the study, they were undergraduate students at Konkuk University, Korea. The norming sentences were split into four lists based on a Latin-square design along with 40 filler sentences with similar complexity. They were pseudo-randomized such that no two sentences from the same condition appeared in a row. Participants were asked to rate the plausibility of the sentences on a scale of 1 (very unlikely) to 5 (very plausible). The rating results were then analyzed using Linear Mixed Effect Regression (LMER) analysis (Baayen, 2008; Baayen et al., 2008; Jaeger, 2008). Models were constructed with the maximal random effect structure and were only simplified when the model did not converge (Barr et al., 2013). The results showed that the plausibility of the four conditions did not significantly differ from each other (|t| < 0.14 for all comparisons: H-NP1 vs. NH-NP2, NH-NP1 vs. NH-NP2, H-NP2 vs. NH-NP2), with the mean ratings of 4.69 (*se*: 0.042), 4.71 (*se*: 0.04), 4.72 (*se:* 0.039), and 4.62 (*se:* 0.046) for the H-NP1 (e.g., *teacher*), NH-NP1 (e.g., *chair*), H-NP2 (e.g., Swujin), and NH-NP2 (e.g., Inho) conditions, respectively.


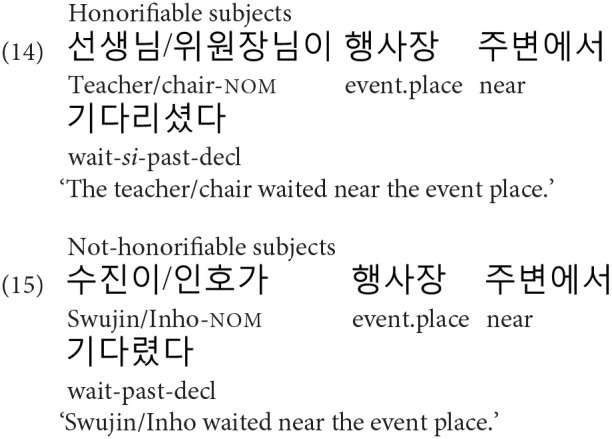


Given the results of the norming study, the experimental stimuli were distributed over four lists based on a Latin square design, along with 80 filler sentences of similar length and complexity. No two experimental items from a same condition were presented in a row.

### Procedure

There were three practice trials before the main experiment started. Participants' eye movements were recorded using an SR Research Eyelink 1,000 Plus eye-tracker at a rate of 1,000 Hz. Each recording session began with a calibration procedure, using a standard 9 point calibration routine before the experiment started, and recalibration was performed whenever necessary throughout the experiment. In each trial, a participant was asked to fixate on a black square on the left side of the screen, where the first character of the upcoming sentence would be presented. When a participant's fixations were successfully detected on the black square, the square was automatically replaced by the experimental stimuli. Participants were asked to read the sentences at their natural speed and answered a yes/no comprehension question for all the sentences. The comprehension questions probed general understanding of the sentences. For example, for the H-H condition sentence in Table 1, “Will the teacher wait around the event place?” was asked. The experiment took about 40 min.

### Data Analysis

For data analysis, following standard eye-tracking data analyses (Rayner, 1998; Sturt, 2007) we first merged short fixations under 80 ms into longer fixations within the distance of the visual angle of 0.05. If there was no such fixation, we removed the short fixations. We also removed fixations longer than 1,200 ms. This procedure affected 1.5% of the trials. Three eye-movement measures are presented. First pass reading times are the sum of all fixations in a target region from the first entry into the region until leaving the region either to the left or right. Go-past times (also called regression path duration) are the sum of all fixations on a given region from the first entry into the region from the left until leaving it to the right. Total time is the sum of all fixations in a given region. For First-pass reading time and Go-past times, we excluded the trials in which a target region was not fixated on in initial reading. For Total Time, we excluded the trials in which the target region received no fixation at all. The proportion of missing data points, due to zeros or track losses were less than 1%.

Statistical analyses were conducted for Region 5 as defined in Table 1, as the region is critical for retrieval processes. The region included the embedded verb and its following adverbial word (mean length = 2.5 syllables). To lower the rate of false positives due to multiple comparisons (von der Malsburg and Angele, 2017), we report statistical analyses for only one region, while reporting means for all regions. We first log-transformed reading times, and the resulting reading time data were analyzed based on Linear Mixed Effect Regression (LMER) analysis (Baayen, 2008; Baayen et al., 2008; Jaeger, 2008), using the lme4 R package (Bates et al., 2015; version 1.1–8). The comprehension accuracy rates were analyzed using a generalized LME model with a binomial distribution. The regression models incorporated two fixed-effect factors (the honorific features of the mrain and the embedded subject: H vs. NH), their interaction and crossed random effects for participants and items. The fixed-effect factors were coded numerically using sum coding, with the two levels of each factor coded as 1 and −1. Models had the maximal random effect structure whenever possible, including both intercepts and slopes, and were only progressively simplified when the model did not converge (Barr et al., 2013). In case of non-convergence, we simplified the model by backwards elimination, following the hierarchy principle, such that the interaction slope parameters were removed, and convergence checked, before attempting to remove either of the random main effect parameters. Also, at each stage of model simplification, convergence was checked both including and excluding random correlation parameters. Random slope parameters corresponding to fixed-effects are reported in in the “slope” column of **Table 4** if they were included in the model for participants or items. Table 4 also shows coefficients, standard errors and *t-*values (*z*-values for the logit model) for each fixed effect and interaction from the analyses. *P-*values were obtained using LmerTest (Kuznetsova et al., 2017), and were corrected for multiple comparisons (9 comparisons: three eye-tracking measures × two main effects and one interaction) using Holm's correction (Holm, 1979; Abdi, 2010). For the analysis of comprehension accuracy based on a binomial logit model, *p*-values were calculated from the Z score, and were also corrected for multiple comparisons (three comparisons: two main effects and one interaction) using Holm's correction. Finally, planned (paired) contrasts were made based on the Tukey test (using the glht function of multcomp package: Hothorn et al., 2008; version 1.4–1) in R (R Core Team, 2018).

### Results and Discussion

Comprehension accuracy and mean reading times for each condition are given in Tables 2, 3, respectively. Statistical analysis results for reading time measures are given in Table 4.

**Table 2:** Mean comprehension accuracy rates in Experiment 1.

	**Mean (se)**		**Estimate**	**SE**	***z***	**Slope**	***p***	**Adjusted *p***
H & H	87.5% (0.019)	Intercept	3.16	0.38	8.22		<2e-16^***^	<2e-16^***^
NH & H	91.8% (0.016)	NP1	−0.14	0.11	−1.29		n.s.	n.s.
H & NH	88.5% (0.019)	NP2	0.07	0.11	0.63		n.s.	n.s.
NH & NH	88.2% (0.019)	NP1^*^NP2	−0.2	0.12	−1.79		0.08	n.s.

**Table 3:** Means (and standard errors), aggregated by participants, for first pass, go-past, and total times in Experiment 1.

	**Region 1 *teacher/swujin***	**Region 2 *chair/Inho***	**Region 3 *event***	**Region 4 *near***	**Region 5 *wait-HON again***	**Region 6 *once***	**Region 7 *emphasized***
**First pass (msec)**							
H & H	417 (24)	373 (14)	254 (7)	244 (7)	711 (23)	275 (10)	299 (15)
NH & H	403 (23)	365 (14)	244 (7)	249 (9)	810 (28)	288 (13)	284 (15)
H & NH	394 (24)	332 (14)	272 (9)	252 (9)	741 (23)	278 (10)	287 (13)
NH & NH	453 (28)	334 (13)	270 (10)	245 (8)	831 (32)	283 (14)	281 (15)
**Go past (msec)**							
H & H		525 (26)	340 (18)	290 (14)	1514 (76)	941 (79)	2484 (169)
NH & H		477 (22)	311 (16)	304 (18)	2007 (111)	1402 (117)	2797 (209)
H & NH		452 (23)	357 (23)	314 (16)	1350 (66)	853 (70)	2004 (113)
NH & NH		458 (21)	369 (25)	330 (20)	1850 (96)	1139 (81)	2541 (182)
**Total time (msec)**							
H & H	855 (38)	1082 (46)	573 (25)	474 (20)	1599 (64)	486 (22)	452 (28)
NH & H	863 (39)	1136 (47)	586 (27)	533 (25)	1925 (83)	596 (27)	432 (30)
H & NH	791 (35)	844 (36)	530 (21)	470 (20)	1462 (56)	483 (22)	398 (20)
NH & NH	903 (38)	982 (51)	623 (33)	501 (23)	1824 (82)	554 (29)	416 (25)

*Grammatical conditions: H & H, H & NH; Ungrammatical conditions: NH & H, NH & NH*.

**Table 4:** Generalized Linear Mixed Effects results for reading times in Experiment 1.

		**Coeff**.	**SE**	***t***	**Slope**	***p***	**Adjusted *p***
**First pass**							
R5 “*wait-HON again”*	Intercept	6.478	0.055	118.94		<2e-16^***^	<2e-16^***^
	NP1	−0.044	0.017	−2.55	(p,i)	0.0116^*^	0.07
	NP2	−0.016	0.02	−0.76	(p,i)	n.s.	n.s.
	NP1^*^NP2	−0.014	0.017	−0.84		n.s.	n.s.
**Go-past**							
	Intercept	7.114	0.089	80.62		<2e-16^***^	<2e-16^***^
	NP1	−0.129	0.026	−5.04	(p,i)	0.00001^***^	0.0001^***^
	NP2	0.032	0.021	1.49	(p,i)	n.s.	n.s.
	NP1^*^NP2	0.009	0.019	0.46		n.s.	n.s.
**Total time**							
	Intercept	7.229	0.0881	82.11		<2e-16^***^	<2e-16^***^
	NP1	−0.093	0.0167	−5.53	(p,i)	0.00001^***^	0.0001^***^
	NP2	0.038	0.0142	2.65	(p,i)	0.0087^*^	0.062+
	NP1^*^NP2	0.005	0.0144	0.28	(p,i)	n.s.	n.s.

*Coefficients, standard errors, t or z-values and p-values are reported for the main effects of NP1and NP2 manipulation, as well as for the interaction of these two factors. Note that the effect of NP1 (the main subject) corresponds to the grammaticality effect, while the effect of NP2 (the dative object) corresponds to the distractor effect. The “Slope” column indicates whether the random slope parameter corresponding to the effect was included in the model for participants (p) or items (i). P-values were obtained using LmerTest (Kuznetsova et al., 2017), and adjusted p values were calculated using Holm's correction for multiple comparisons (Holm, 1979; Abdi, 2010)*.

* *p < 0.05*,

*** *p < 0.0005*.

Region 5 (the critical embedded verb and the spill over region; ‘wait-hon again')

At R5, there was a main effect of NP1 with the (ungrammatical) NH_NP1_ conditions taking longer to read than the (grammatical) H_NP1_ conditions. The effect was marginal in First pass times and significant in Go-past and Total times. In addition, there was a marginal main effect of NP2 in Total times with longer reading times for the H_NP2_ condition than for the NH_NP2_ condition.

The grammaticality effect (i.e., the main effect of NP1) suggests that the subject control information was accessed from an early processing stage, allowing, and constraining dependency formation between the verb and its licit subject throughout the various processing stages. On the other hand, the marginal effect of NP2 in Total times suggests a tendency for a distractor with matching features to be activated regardless of grammaticality of the target sentences. For both grammatical and ungrammatical sentences, a distractor matching the verb in the honorific feature led to a slow-down in Total Time, relative to conditions where the distractor mismatched, presumably reflecting a later stage of processing.

The grammaticality effect shows that readers were sensitive to the relevant control information in forming the dependency. On the other hand, the (marginal) effect of the distractor was different from the so-called “attraction effect,” where facilitation is limited to the ungrammatical conditions (Wagers et al., 2009). Instead, the direction of the effect was such that the distractor interfered with and slowed down reading, when it matched the honorific features of the verb, regardless of the grammaticality of the sentence. We note that this effect is different from the general pattern observed by Kwon and Sturt (2016b), where a matching distractor tended to facilitate processing. We return to this point in the general discussion.

In summary, in Experiment 1 the grammaticality effect preceded any effect of the distractor. That is, while the grammaticality effect was found both in Go-past and Total times on the critical verb, the effect of the distractor was found only in Total Time. In addition, the marginal main effect of the distractor suggests that the distractor may affect the processing of the subject control construction regardless of grammaticality of the target sentences.

## Experiment 2

Experiment 2 investigated the processing of subject-verb honorific agreement in the object control construction with –*la* in Korean. The embedded verb marked with –*la* signals that the indirect object (NP2) marked with a dative case marker is the licit controller for PRO, as shown in (16). Thus, the dependency is formed between the dative marked NP2 and the embedded verb (via PRO), as illustrated in (11) and (16). On the other hand, the nominative marked main clause subject (NP1) cannot serve as a controller.

(16) Object control construction used in Experiment 2
[NP1_*i*_-nom NP2*_*k*_*-dat
[PRO*_*k*_* leave-hon-object.ctrl-comp] said]
‘NP_*i*_ told NP2_*k*_ to PRO${}_{{}^{*}i/k}$ leave.'

As in Experiment 1, we predicted that mismatched honorific features of the incongruous conditions with the NH subject (NH dative object conditions: H_NP1_-NH_NP2_ & NH_NP1_-NH_NP2_) would incur processing difficulty (Kwon and Sturt, 2016b), leading to longer reading times at the critical region in these conditions than in the congruous conditions (H-H & NH-H conditions). In addition to the grammaticality effect, we also predicted an effect of the distractor. In particular, if interference from a distractor is affected by memory representations of a distractor, it is likely that a memory representation that is more highly activated at the point of retrieval will be more likely to lead to interference. If so, we predict that the effect of a distractor will be weaker in Experiment 2 than in Experiment 1, given that the distractor position is further away from the embedded verb and so its memory representation could be more decayed at the retrieval point in Experiment 2.

### Participants

Twenty eight native speakers of Korean (mean age: 23.96; range 19–31) participated in the study, receiving KRW 10,000 per hour. They were all either undergraduate or graduate students at Konkuk University, Korea, and had normal or corrected-to-normal vision. Written informed consent was obtained from all the participants.

### Materials

Forty sets of object control sentences like those in Table 5 were created based on the stimuli of Experiment 1, replacing the subject control suffix—*keyss* with the object control suffix –*la*. Lexical items remained the same as in Experiment 1, but main verbs were changed when necessary. Thus, at the point of the embedded verb, the plausibility of the target sentences with potential subject NPs was identical to that in Experiment 1. We used the same filler sentences used in Experiment 1, and other remaining procedures were also analogous to Experiment 1.

**Table 5:**
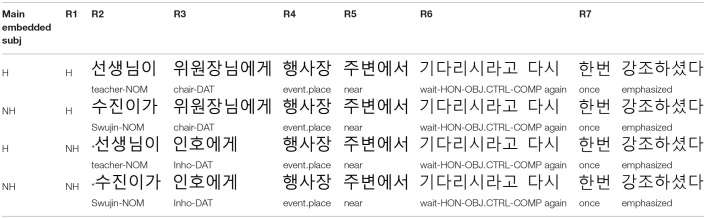
Example experiment item in Experiment 2: Object control construction.

*The teacher/Swujin emphasized to the chair_i_/Indo_i_ that ___i_ should wait near the event place*.

### Procedures

The same eye-tracking procedure was used as in Experiment 1.

### Data Analysis

As in Experiment 1, we first merged short fixations under 80 ms into longer fixations within the distance of the visual angle of 0.05. On the other hand, fixations longer than 1,200 ms were removed. This procedure affected 2.1% of the trials. On the other hand, the proportion of missing data points, due to zeros or track losses were <2%. Remaining procedures were analogous to those used in Experiment 1.

### Results and Discussion

Mean comprehension accuracy and reading times for each condition are given in Tables 6, 7, respectively, and statistical analysis results are given in Table 8.

**Table 6:** Mean comprehension accuracy rates in Experiment 2.

	**Mean (se)**		**Estimate**	**SE**	***z***	**Slope**	***p***	**Adjusted *p***
H & H	89.3% (0.018)	Intercept	3.27	0.39	8.36		<2e-16^***^	<2e-16^***^
NH & H	90% (0.017)	NP1	−0.019	0.11	−0.17		n.s.	n.s.
H & NH	90% (0.017)	NP2	0.004	0.11	0.04		n.s.	n.s.
NH & NH	89.3% (0.018)	NP1^*^NP2	0.046	0.11	−0.41		n.s.	n.s.

**Table 7:** Means (and standard errors), aggregated by participants, for first pass, go-past, and total times in Experiment 2.

	**Region 1*teacher/swujin***	**Region 2*chair/Inho***	**Region 3*event***	**Region 4*near***	**Region 5*wait-HON again***	**Region 6*once***	**Region 7*emphasized***
**First pass (msec)**							
H & H	364 (23)	328 (12)	256 (7)	221 (7)	700 (24)	256 (9)	313 (19)
NH & H	340 (16)	357 (13)	250 (7)	240 (7)	776 (25)	277 (10)	290 (20)
H & NH	345 (17)	313 (11)	267 (11)	232 (8)	750 (25)	280 (11)	330 (22)
NH & NH	373 (26)	318 (11)	260 (8)	239 (8)	771 (24)	284 (13)	288 (24)
**Go past (msec)**							
H & H		468 (22)	319 (15)	315 (22)	1274 (58)	729 (58)	1921 (119)
NH & H		457 (18)	328 (17)	323 (21)	1447 (64)	832 (57)	1924 (136)
H & NH		445 (20)	323 (16)	287 (15)	1447 (66)	762 (60)	2324 (142)
NH & NH		447 (21)	319 (16)	343 (24)	1640 (72)	1001 (70)	1855 (126)
**Total time (msec)**							
H & H	753 (36)	880 (35)	508 (21)	441 (21)	1281 (50)	415 (19)	415 (23)
NH & H	649 (30)	894 (37)	480 (19)	419 (19)	1351 (47)	471 (23)	440 (31)
H & NH	782 (38)	874 (31)	561 (24)	453 (19)	1538 (57)	493 (23)	488 (27)
NH & NH	737 (37)	843 (34)	523 (21)	431 (19)	1370 (49)	476 (25)	373 (31)

*Grammatical conditions: H & H, NH & H; Ungrammatical conditions: H & NH, NH & NH*.

**Table 8:** Generalized Linear Mixed Effects results for reading times in Experiment 2.

		**Coeff**.	**SE**	***t***	**Slope**	***p***	**Adjusted *p***
**First pass**							
R5 “*wait-HON again”*	Intercept	6.485	0.056	116.76		<2e-16^***^	<2e-16^***^
	NP1	−0.043	0.018	−2.39^*^	(p,i)	0.0238	n.s.
	NP2	−0.014	0.016	−0.87	(p,i)	n.s.	n.s.
	NP1*NP2	−0.011	0.016	−0.69	(p,i)	n.s.	n.s.
**Go-past**							
	Intercept	7.054	0.057	122.81		<2e-16^***^	<2e-16^***^
	NP1	−0.067	0.02	−3.35^*^	(p,i)	0.0019	0.0172^*^
	NP2	−0.054	0.024	−2.3^*^	(p,i)	0.027	n.s.
	NP1^*^NP2	0.006	0.022	0.29	(p,i)	n.s.	n.s.
**Total time**							
	Intercept	7.069	0.067	105.51		<2e-16^***^	<2e-16^***^
	NP1	0.001	0.014	0.08	(p,i)	n.s.	n.s.
	NP2	−0.05	0.016	−3.2^*^	(p)	0.003	0.0235^*^
	NP1*NP2	−0.042	0.014	−3.07^*^		0.002	0.0177^*^

*Coefficients, standard errors, t or z-values and p-values are reported for the main effects of NP1 and NP2, as well as for the interaction of these two factors. Note that the effect of NP2 (the dative object) corresponds to the grammaticality effect, while the effect of NP1 (the main subject) corresponds to the distractor effect. The “Slope” column indicates whether the random slope parameter corresponding to the effect was included in the model for participants (p) or items (i). P-values were obtained using LmerTest (Kuznetsova et al., 2017) and adjusted p-values were calculated using Holm's correction for multiple comparisons (Holm, 1979; Abdi, 2010)*.

* *p < 0.05*.

Region 5 (the critical embedded verb and the spill over region; ‘wait-hon again')

At R5, there was a main effect of the NP1 (main subject: distractor) in Go-past times with longer reading times for the NH_NP1_ conditions, where the distractor mismatches the honorific features of the verb (i.e., NH_NP1_-H_NP2_ & NH_NP1_-NH_NP2_) than for the H_NP1_ conditions, where it matches (i.e., H_NP1_-H_NP2_ & H_NP1_-NH_NP2_). In addition, there was also a main effect of NP2 (dative marked object NP: licit subject for the embedded verb) in Total times with longer reading times for the ungrammatical NH_NP2_ conditions (i.e., H_NP1_-NH_NP2_ & NH_NP1_-NH_NP2_) than for the grammatical H_NP2_ conditions (i.e., H_NP1_-H_NP2_ & NH_NP1_-H_NP2_). In Total time, there was also an interaction of NP1 and NP2. *Post-hoc* pairwise comparisons showed that this was because reading times to the H_NP1_-NH_NP2_ condition were significantly longer than those to the H_NP1_-H_NP2_ condition (*p* < 0.001), reflecting a grammaticality cost when the distractor matched the honorific feature of the verb. On the other hand, reading times to the NH_NP1_-NH_NP2_ condition were not significantly different from those to NH_NP1_-H_NP2_ condition (n.s.), reflecting the lack of a grammaticality cost in this measure when the distractor mismatched the honorific feature of the verb. Note that the form of this interaction is different from what would be expected based on previous literature on subject-verb agreement attraction in English (e.g., Wagers et al., 2009; Dillon et al., 2013), where the grammaticality cost is typically found to be *reduced* by a matching distractor.

In summary, these results suggest that the processing of object control construction was not only affected by a licit dative object controller (NP2) but also by a structurally illicit subject controller (i.e., NP1). In addition, the effect of a distractor preceded the grammaticality effect and was detected from a relatively earlier eye-gazing measurement (Go-past times), and its effect was not limited to ungrammatical sentences.

## Experiment 3

Experiment 3 investigated the processing of subject-verb honorific agreement in the center-embedded construction, where an embedded clause serves as a sentential complement of the main verb. While both NP1 and NP2 are marked with a nominative case marker, the embedded verb in this construction signals that the embedded subject NP (NP2 in this case) is its licit subject, as shown in (12) and in (17).

(17) Center embedded construction used in Experiment 3
[main cl. NP1-nom        [embedded cl. NP2-nom
leave-hon-pst-decl-comp]    said]
‘NP1 said that NP2 left.'

As in Experiments 1 and 2, we predicted that the incongruous conditions with the NH subject (H_NP1_-NH_NP2_ & NH_NP1_-NH_NP2_ conditions) would show longer reading times at the critical region than in the congruous conditions (H-H & NH-H conditions). In addition, assuming that case information is a retrieval cue (cf. Hartsuiker et al., 2001), the distractor in Experiment 3 matches more retrieval cue features than the distractor in Experiments 1 or 2. Thus, if a greater degree of feature overlap between distractor and the retrieval cues leads to greater interference or attraction, then Experiment 3 is predicted to show a stronger effect of a distractor than Experiment 2.

### Participants

Twenty eight native speakers of Korean (mean age: 23.89; range 21–31) received KRW 10,000 and participated in the study. They all had normal or corrected-to-normal vision, and attended Konkuk University, Korea at the time of the experiment. Written informed consent was obtained from all the participants.

### Materials

Forty sets of center-embedded complement sentences like those in Table 9 were created. As in Experiment 2, there was no change in lexical items before the main verb position. Thus, at the critical region (i.e., the embedded verb region) the plausibility of the target sentences with potential subject NPs remained the same as in Experiments 1 and 2. In addition, as in Experiment 2, the same filler sentences used in Experiment 1 were employed. Remaining procedures were also analogous to Experiments 1 and 2.

**Table 9:**
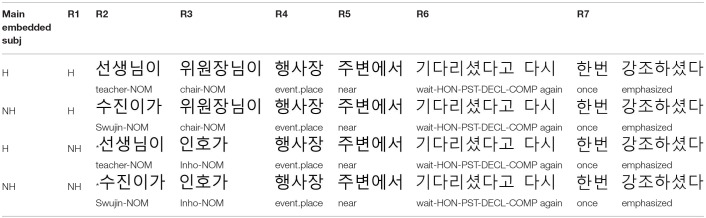
Example experiment item in Experiment 3: Center-embedded construction.

*The teacher/Swujin emphasized to the chair_i_/Indo_i_ that ___i_ should wait near the event place*.

### Procedures

The same eye-tracking procedure was used as in Experiments 1 and 2.

### Data Analysis

As in Experiments 1 and 2, short fixations under 80 ms were first merged into longer fixations within the distance of the visual angle of 0.05. Then, we removed fixations longer than 1,200 ms. This procedure affected 2.2% of the total trials. On the other hand, the proportion of missing data points, due to zeros or track losses were <1%. Analogous statistical analysis procedures were applied as in Experiments 1 and 2.

### Results and Discussion

Mean comprehension accuracy and reading times for each condition are given in Tables 10, 11, respectively, and statistical analysis results are given in Table 12.

**Table 10:** Mean comprehension accuracy rates in Experiment 3.

	**Mean (se)**		**Estimate**	**SE**	***z***	**Slope**	***p***	**Adjusted *p***
H & H	93.2% (0.015)	Intercept	3.926	0.46	8.44		<2e-16^***^	<2e-16^***^
NH & H	92.5% (0.015)	NP1	0.229	0.13	1.71		0.09	n.s.
H & NH	95% (0.013)	NP2	−0.037	0.13	−0.28		n.s.	n.s.
NH & NH	91.1% (0.017)	NP1^*^NP2	−0.161	0.13	−1.21		n.s.	n.s.

**Table 11:** Means (and standard errors), aggregated by participants, for first pass, go-past, and total times in Experiment 3.

	**Region 1 *teacher/swujin***	**Region 2 *chair/Inho***	**Region 3 *event***	**Region 4 *near***	**Region 5 *wait-HON again***	**Region 6 *once***	**Region 7 *emphasized***
**First pass (msec)**							
H & H	393 (20)	327 (12)	258 (7)	238 (7)	700 (27)	265 (9)	333 (19)
NH & H	376 (17)	340 (13)	269 (8)	245 (8)	737 (26)	273 (11)	295 (16)
H & NH	391 (20)	312 (14)	260 (8)	229 (7)	728 (24)	261 (8)	331 (18)
NH & NH	356 (18)	316 (11)	257 (8)	241 (8)	809 (28)	271 (10)	298 (15)
**Go past (msec)**							
H & H		521 (26)	488 (27)	406 (26)	1279 (63)	731 (60)	1543 (98)
NH & H		489 (20)	407 (20)	311 (15)	1385 (65)	824 (55)	1791 (133)
H & NH		431 (21)	388 (19)	390 (27)	1381 (69)	785 (68)	1882 (117)
NH & NH		566 (31)	496 (26)	376 (24)	1757 (93)	1104 (100)	1978 (137)
**Total time (msec)**							
H & H	729 (31)	839 (34)	558 (20)	431 (19)	1226 (47)	401 (19)	448 (26)
NH & H	685 (32)	821 (34)	567 (23)	453 (23)	1280 (49)	444 (21)	408 (26)
H & NH	719 (33)	728 (31)	605 (24)	465 (21)	1375 (52)	457 (21)	501 (29)
NH & NH	825 (41)	899 (35)	677 (30)	465 (22)	1521 (53)	471 (23)	425 (24)

*Grammatical conditions: H & H, NH & H; Ungrammatical conditions: H & NH, NH & NH*.

**Table 12:** Generalized Linear Mixed Effects results for reading times in Experiment 3.

		**Coeff**.	**SE**	***t***	**Slope**	***p***	**Adjusted *p***
**First pass**							
R5 “*wait-HON again”*	Intercept	6.455	0.045	142.67		<2e-16^***^	<2e-16^***^
	NP1	−0.04	0.019	−2.13^*^	(p,i)	0.043	n.s.
	NP2	−0.039	0.017	−2.27^*^	(p,i)	0.029	n.s.
	NP1^*^NP2	0.004	0.016	0.26		n.s.	n.s.
**Go-past**							
	Intercept	7.021	0.066	106.48		<2e-16^***^	<2e-16^***^
	NP1	−0.081	0.024	−3.42^*^	(p,i)	0.0018	0.014^*^
	NP2	−0.072	0.02	−3.53^*^	(p,i)	0.0015	0.013^*^
	NP1^*^NP2	0.03	0.019	1.59	(p,i)	n.s.	n.s.
**Total time**							
	Intercept	7.041	0.071	99.11		<2e-16^***^	<2e-16^***^
	NP1	−0.04	0.017	−2.33^*^	(p,i)	0.026	n.s.
	NP2	−0.077	0.016	−4.92^*^	(p,i)	0.0001	0.0004^***^
	NP1^*^NP2	0.015	0.015	1.05	(p,i)	n.s.	n.s.

*Coefficients, standard errors, t or z-values and p-values are reported for the main effects of NP1 and NP2 manipulation, as well as for the interaction of these two factors. Note that the effect of NP2 (the embedded subject) corresponds to the grammaticality effect, while the effect of NP1 (the main subject) corresponds to the distractor effect. The “Slope” column indicates whether the random slope parameter corresponding to the effect was included in the model for participants (p) or items (i). P-values were obtained using LmerTest (Kuznetsova et al., 2017) and adjusted p-values were calculated using Holm's correction for multiple comparisons (Holm, 1979; Abdi, 2010)*.

* *p < 0.05*,

*** *p < 0.0005*.

Region 5 (the critical embedded verb and the spill over region; ‘wait-hon again')

At R5, there was a main effect of the NP1 (distractor effect) and NP2 (grammaticality effect) in Go-past times, and a main effect of NP2 in Total times. The pattern was similar to that seen in Experiment 2. Both NH_NP1_ and NH_NP2_ conditions elicited longer reading times than their counterpart H_NP1_ and H_NP2_ conditions, respectively, reflecting a cost for the mismatching of honorific features between the NP and the verb. These effects suggest that the processing of honorific agreement in the embedded verb is affected both by a licit (NP2) and illicit (NP1) subject. There was, however, no significant interaction between the two.

In summary, the processing of the center-embedded construction was affected by both NP1 (distractor) and NP2 (the licit subject), and the effect of a distractor was not limited to ungrammatical sentences. In addition, while both the grammaticality effect and the distractor effect were detected in the same early eye-tracking measure (Go-past times), only the grammaticality effect was found in Total times, which are a more general measure of processing.

## General Discussion and Conclusion

The goal of the study was to investigate how memory representations of a distractor affect the retrieval processes. In particular, we were interested in the effect of proximity of a distractor to a retrieval point. Our reasoning was that if a distractor is temporarily closer to a retrieval point, then its memory representation is more likely to be highly activated, and thus is more likely to lead to stronger interference or attraction than when it is further away from the retrieval point. Thus, we predicted a stronger interference effect in the subject control construction (10) than in the object control construction (11). In addition, we aimed to examine whether a distractor would lead to stronger interference when there is a higher degree of feature match between the distractor and the retrieval cues. If a distractor is activated based on feature matches with retrieval cues, then it is possible that multiple feature matches could lead to stronger activation of the distractor, leading to a stronger interference effect. Thus, we predicted a stronger interference effect in the center-embedded construction (12) than in the object control construction (11). To address these questions, we directly compared the results of Experiments 1 and 2, and the results of Experiments 2 and 3.

We re-analyzed Go-past times of the critical region (Region 5 in Experiments 1–3), including Experiment as a fixed-effect factor in the models. In addition, since the licit subject differs for Experiment 1 (i.e., NP1) and Experiments 2 and 3 (i.e., NP2), NP1 and NP2 were re-coded as the licit or the illicit (distractor) subject, and were also incorporated in the regression models as such, so that the effect of a distractor can be better compared across the experiments. The remaining procedures were analogous to those reported in Experiments 1–3. The results of the statistical analyses comparing the results of Experiments 1 and 2 on the one hand, and the results of Experiments 2 and 3 on the other are presented in Tables 13, 14, respectively. *P*-values were corrected for multiple comparisons using Holm's correction (Holm, 1979; Abdi, 2010).

**Table 13:** Generalized Linear Mixed Effects results comparing Go-past times of Experiments 1 and 2.

	**Coeff**.	**SE**	***t***	**Slope**	***p***	**Adjusted *p***
(Intercept)	7.083	0.055	129.62		<2e-16^***^	<2e-16^***^
Licit NP	−0.092	0.013	−6.95^*^		4.78e-12^***^	3.346e-11^***^
Illicit NP	−0.019	0.013	−1.42		n.s.	n.s.
Experiments	−0.03	0.051	−0.59		n.s.	n.s.
Licit^*^Illicit	0.007	0.013	0.57		n.s.	n.s.
Licit^*^Experiments	0.036	0.013	2.73		0.0064^*^	0.032^*^
Illicit^*^Experiments	−0.05	0.013	−3.77		0.00016^***^	0.001^**^
Licit^*^Illicit^*^Experiments	−0.001	0.013	−0.09		n.s.	n.s.

*Coefficients, standard errors, t or z-values and p-values are reported for the main effects of the licit NP, the illicit NP and Experiment, as well as for the interactions of these three factors. Note that the effect of the illicit NP corresponds to the grammaticality effect, while the effect of illicit NP corresponds to the distractor effect. The “Slope” column indicates whether the random slope parameter corresponding to the effect was included in the model for participants (p) or items (i). P-values were obtained using LmerTest (Kuznetsova et al., 2017) and adjusted p-values were calculated using Holm's correction for multiple comparisons (Holm, 1979; Abdi, 2010)*.

* *p < 0.05*,

** *p < 0.005*,

*** *p < 0.0005*.

**Table 14:** Generalized Linear Mixed Effects results comparing Go-past times of Experiments 2 and 3.

	**Coeff**.	**SE**	***t***	**Slope**	***p***	**Adjusted *p***
(Intercept)	7.037	0.045	155.19		<2e-16^***^	<2e-16^***^
Licit NP	−0.075	0.015	−4.99	(p, i)	0.00003^***^	0.00021^***^
Illicit NP	−0.064	0.015	−4.22	(i)	0.00015^***^	0.0011^**^
Experiments	−0.015	0.042	−0.36		n.s.	n.s.
Licit^*^Illicit	0.017	0.013	1.36		n.s.	n.s.
Licit^*^Experiments	−0.007	0.013	−0.53		n.s.	n.s.
Illicit^*^Experiments	−0.009	0.013	−0.69		n.s.	n.s.
Licit^*^Illicit^*^Experiments	0.013	0.013	0.99		n.s.	n.s.

*Coefficients, standard errors, t or z-values and p-values are reported for the main effects of the licit NP, the illicit NP and Experiment, as well as for the interactions of these three factors. Note that the effect of the illicit NP corresponds to the grammaticality effect, while the effect of illicit NP corresponds to the distractor effect. The “Slope” column indicates whether the random slope parameter corresponding to the effect was included in the model for participants (p) or items (i). P-values were obtained using LmerTest (Kuznetsova et al., 2017) and adjusted p-values were calculated using Holm's correction for multiple comparisons (Holm, 1979; Abdi, 2010)*.

** *p < 0.005*,

*** *p < 0.0005*.

The comparison of Experiments 1 and 2 showed a main effect of Licit NP (*p* < 0.0001), but the effect was accompanied by a significant interaction of Licit NP and Experiment (*p* < 0.032). The main effect suggests that the processing of honorific agreement is constrained by honorific features of the licit subject both in the subject control (Experiment 1) and the object control (Experiment 2) constructions, but the interaction suggests that the magnitude of grammaticality effect varies by the function of the Experiment. Indeed, the grammaticality effect survived Holm's adjustment for multi-comparisons only in Experiment 1 (NP1 effect in Table 4; adjusted *p* < 0.0001) but not in Experiment 2 (NP2 effect in Table 8; adjusted *p* = n.s.), suggesting that the grammaticality effect in Experiment 2 was relatively weaker than that in Experiment 1. On the other hand, there was no main effect of Illicit NP, but there was a significant interaction of Illicit NP and Experiment (*p* < 0.001). This seems to be due to a significant Illicit NP effect in Experiment 2 (NP1 effect in Table 8; adjusted *p*-value < 0.017) but not in Experiment 1 (NP2 effect in Table 4; adjusted *p* = n.s.), suggesting that the Go-past reading times for honorific agreement were affected by an illicit NP in the object control construction but not in the subject control construction.

On the other hand, the analyses comparing Experiments 2 and 3 showed main effects of both Licit NP (*p* < 0.0003) and Illicit NP (*p* < 0.002), but there was no significant interaction with Experiment. Thus, we do not have evidence for a difference in the effect of the licit NP in the object control and the embedded clause construction, despite the fact that the significant grammaticality effect survived Holm's adjustment for multi-comparisons in Experiment 3 (NP2 effect in Table 12; adjusted *p* < 0.014) but not in Experiment 2. Likewise, there was no interaction of Illicit NP with Experiment, suggesting that the processing honorific –*si* dependency is sensitive to the properties of the illicit NP in the object control and embedded clause construction to a similar degree.

Overall, these results showed that (i) the effect of a licit NP was stronger in the subject control than in the object control construction, (ii) the effect of an illicit NP was stronger in the object control than in the subject control, and (iii) the effect of a licit NP and an illicit NP was found to a similar degree in the object control and the embedded clause construction. We address the implications of these findings in turn below.

First, the observations (i) and (ii) above suggests that the proximity of a distractor to a retrieval point does not modulate the attraction effect. While a marginal distractor effect was also found in the subject control construction (Experiment 1), the effect was only observed in the Total reading times, probably reflecting relatively late processing. In addition, we suspect that the distractor effect in Experiment 1 may have been spurious, given that it was inhibitory, while the overall pattern for the distractor effect was facilitatory in Experiments 2 and 3, as well as in the experiments reported by Kwon and Sturt (2016b). We therefore reserve judgment on the status of the distractor effect in Experiment 1, pending replications in further research. On the other hand, there are several possibilities why the distance manipulation did not affect the interference effect in the study. Previous studies have shown the effect of a temporal (or linear) distance during on-line sentence processing (Warren and Gibson, 2002). While it has been controversial, the claim is that when a linear distance is shorter between two linguistic items, they are easier to integrate together than when the distance between the two is longer (for details of such proposals, see Gibson, 1998, 2000; Lewis and Vasishth, 2005; Lewis et al., 2006; for a review, see Kwon et al., 2010). On the other hand, the results from the current study suggest that linear distance does not affect sentence processing during retrieval processes. Instead, it could be the case that as long as a distractor is re-activated above a certain threshold level during a retrieval process, it interferes with the processing of a subject-verb dependency, and the interference effect is not further modulated by the level of activation of the distractor at the point of retrieval. In fact, it should be also noted that there was no evidence that the linear distance affected the integration of the licit subject and its verb either. If the linear distance affected the integration difficulty, the subject control construction should have elicited longer reading times than the object control construction due to longer linear/temporal distance between the licit subject and the verb. There was, however, no evidence that integration of the licit subject and the verb was more difficult in the subject control construction (Experiment 1) than in the object control construction (Experiment 2). Thus, the overall results suggest that at least for the construction examined here, temporal (or linear) distance is not a factor affecting sentence processing during the retrieval (or integration) processes. Alternatively, it is possible that temporal (or linear) distance affects the retrieval process, but there are other factors, which are more important than the mere linear distance difference to affect interference effect, and thus the effect of the linear distance is overridden by other factors of importance. It is, of course, also possible that our design was not powerful enough to detect such differences. Our data do not distinguish between these possibilities, but we will return to this issue below.

As the observation (iii) above indicates, the results also did not provide evidence that the degree of feature overlap between a distractor and retrieval cues affected the interference effect of a distractor. The effects of the distractor were of a similar size in the two experiments, and showed the same facilitatory direction of effect. This contrasts with Parker and Phillips (2017), where a distractor associated with a larger number of matching features significantly affected the processing of a dependency involving a reflexive. However, unlike in our study, Parker and Phillips also manipulated retrieval cues of a licit target in addition to those of a distractor, and found the effect of a distractor when the number of matching features was reduced for the licit target but increased for the distractor. This suggests that interference from a distractor is not just sensitive to the memory representation of a distractor, but also to activation of a distractor relative to that of a target.

On the other hand, lack of clear evidence in support of the case marker as a retrieval cue seems surprising given previous studies. For example, in a production study in Dutch Hartsuiker et al. (2001) showed that the attraction effect diminished when case marking of a distractor was clearly distinct from that of the licit subject, but increased when the case of the distractor was ambiguous. Likewise, importance of case information as a retrieval cue has been also discussed in several comprehension studies. For example, Van Dyke and McElree (2011) reported stronger interference effects when a distractor (e.g., *the neighbor*) appeared in a structurally similar position to that of the licit subject (e.g., *the worker* and *the resident* in “The worker was surprised that the resident who said the neighbor was dangerous was complaining about the investigation”) than when a distractor (e.g., *the witness*) appeared in a structurally different position (e.g., the attorney *and the judge* in “The attorney who the judge realized had rejected the witness in the case compromised”). Similar results were also reported in Arnett and Wagers (2017). After examining the processing of subject-verb agreement in sentential complement, ECM, and object control construction sentences, Arnett and Wagers argued that the interference effect is modulated by the structural position as well as case properties of a distractor. All these results suggest that structural information (i.e., whether an NP is a subject or an object) is an important retrieval cue, but this argument was not confirmed in the current study. However, it should be noted that the clear interference effects reported in Van Dyke and McElree (2011) and Arnett and Wagers came from additional manipulations of comprehension difficulty, either involving semantic relatedness (Van Dyke and McElree, 2011) or semantic complexity (Arnett and Wagers). In contrast, reading time results of those studies were not straightforward. For example, Arnett and Wagers found different reading time results only for the comparison of sentential complement constructions with ECM construction, but no difference was found for the comparisons of object control constructions with other constructions. Given this, our results are not incompatible with these previous studies.

We consider how our results fit with the predictions of the cue-based retrieval model as proposed by Lewis and Vasishth (2005). The most straightforward version of this model predicts a small inhibitory effect of a matching (relative to a mismatching) distractor in grammatical conditions, and a larger facilitatory effect of a matching distractor in ungrammatical conditions. The reason for the inhibitory prediction in the grammatical conditions is that the partial match of the distractor with the retrieval cues decreases the activation of the target of the dependency, leading to a prediction of more processing difficulty, relative to a case where the distractor does not match in any features. In contrast, in ungrammatical conditions, the fact that both target and distractor partially match the retrieval cues leads to the distractor being mis-retrieved in a proportion of trials, leading to shorter average processing times (i.e., facilitation), relative to the situation where the distractor completely mismatches the retrieval cues.

Given these considerations, the Lewis and Vasishth (2005) model would predict an interaction between the matching of target and distractor NPs in our experiments. Specifically, for the grammatical conditions, where the target NP matches the honorific features of the verb, the model would have predicted longer reading times where the distractor also matches the honorific features, relative to when it does not. In contrast, for the ungrammatical conditions, where the target NP does not match the honorific features of the verb, the model would have predicted facilitation where the distractor is honorific, relative to when it is not. However, this specific form of interaction was not found in our experiments. Instead, in Experiments 2 and 3, we found a general effect of facilitation, which in most measures did not significantly differ between grammatical and ungrammatical conditions, while in Experiment 1, if anything, the evidence suggests a general inhibitory effect of the distractor, again not interacting with the matching of the licit NP subject.

On the basis of a large-scale meta-analysis, Jäger et al. (2017) point out that several studies of attraction in verb-subject agreement show facilitation for matching distractors, even in grammatical conditions, as we found in our Experiments 2 and 3, but contra the predictions of Lewis and Vasishth (2005). One suggestion that has recently been made by Engelmann et al. (2019) is that the direction of the distractor effect in grammatical conditions may depend on the relative prominence of the target of the dependency and the distractor. In Engelmann et al.'s extended version of the Lewis and Vasishth (2005) model, the prominence of the distractor affects its baseline activation, leading to a prediction of facilitatory interference in grammatical conditions, where the distractor is particularly prominent, for example when the distractor is a main clause subject. In fact, we believe that our experimental results are consistent with this prediction. The distractor in both of Experiments 2 and 3 was a main clause subject, and we found robust evidence of a distractor effect in both of the experiments, with a general facilitatory effect of the distractor. In contrast, when a distractor was a dative argument with low prominence, and when the licit target of the dependency is the main clause subject as in our Experiment 1, there was no clear effect of a distractor. These results suggest that relative prominence of distractor in comparison to that of a target could affect the retrieval processes during the processing of a dependency. If so, it is also possible that the relative prominence of distractor could have over-ridden any effect that a linear distance could have.

On the other hand, we believe that prominence of a distractor could vary depending the nature of a dependency. It is likely that a main clause subject is particularly more prominent than a dative marked object when processing a subject-verb dependency. It is an empirical question whether similar levels of prominence will be observed for a main clause subject or an object when a dependency is not relevant for subjecthood, for example, as in the case of the processing of object-verb agreement. That is, it is suggested that individual languages might differ in the relative weight assigned to various sources of information used for language processing, and that this could be due to typological variations in the importance of those cues (Kwon and Sturt, 2013). Given this, it is also a possibility that cues employed for language processing are weighted in a given context. In other words, different levels of prominence could be associated with different cues in a different context. However, future research should examine the effect of distractor prominence more systematically.

Finally, the results of the current study are not compatible with the hypothesis that the attraction effect is an error-driven processing mechanism (Pearlmutter et al., 1999; Wagers et al., 2009; Dillon et al., 2013; Lago et al., 2015; cf. Van Dyke, 2007). First, attraction effect was not found just for ungrammatical sentences but also for grammatical sentences. Second, in Experiment 2, attraction effect even preceded grammaticality effect. These results are consistent with previous findings of honorific agreement in Korean, supporting the view that attraction effect arises from general working memory principles (Kwon and Sturt, 2016b). That is, during the processing of a dependency, items with matching retrieval features are activated, even the feature match is only partial, affecting the processing of the dependency.

In summary, the current study investigated whether and how attraction effects would be modulated by the memory representation of a distractor, by examining the subject-verb honorific agreement in Korean. Our study did not find evidence that proximity of a distractor to the retrieval point (i.e., higher activation level of a distractor) increased interference effects of the distractor. Similarly, we did not find evidence that a higher number of matching cues of a distractor triggered more mis-retrieval. Instead, the results suggested that interference is not just sensitive to memory representation of a distractor but rather to activation of a distractor relative to that of a target. Our results are also consistent with Engelmann et al. (2019)'s proposal that the prominence of the distractor affects the direction of the distractor effect in grammatical conditions.

## Data Availability

The datasets generated for this study are available on request to the corresponding author.

## Ethics Statement

We obtained full IRB approval for the study from the Human Research Protections Programs at Konkuk University.

## Author Contributions

NK and PS were both involved in the design of the study, and prepared the manuscript. NK carried out the experiment and analyzed the data.

### Conflict of Interest Statement

The authors declare that the research was conducted in the absence of any commercial or financial relationships that could be construed as a potential conflict of interest.

## Footnotes

^1^Our predictions are based on Lewis and Vasishth (2005), which assumes time-based decay in working memory. While it should be noted that the notion of temporal decay has been controversial, with a possibility that time-based forgetting could be due to representation-based interference (Nairne, 2002; Oberauer and Kliegl, 2006; Lewandowsky et al., 2009) rather than time-related decay (Berman et al., 2009; Barrouillet et al., 2011), for our experiment, the interference-based account also makes the same prediction with the time-based decay account, given that a distractor intervenes with the subject-verb agreement in the subject control construction but not in the object control construction.

^2^We intentionally did not use non-honorific description nouns (e.g., baby) for the NH conditions. This is because two consecutively occurring description NPs can be misanalysed as an instance of a double nominative construction in Korean, where the NP2 can inherit the honorific features of the NP1 and thus can be predicated with a honorific verbal form even when NP2 is normally not honorifiable (Sohn, 1999; e.g., *Teacher-nom house-nom is.far-**si-decl*, “The teacher, his place is far away”).
